# How integration of global omics-data could help preparing for pandemics – a scent of influenza

**DOI:** 10.3389/fgene.2014.00080

**Published:** 2014-04-22

**Authors:** Lieuwe D. J. Bos, Menno D. de Jong, Peter J. Sterk, Marcus J. Schultz

**Affiliations:** ^1^Department of Intensive Care Medicine, Academic Medical Center, University of AmsterdamAmsterdam, Netherlands; ^2^Department of Respiratory Medicine, Academic Medical Center, University of AmsterdamAmsterdam, Netherlands; ^3^Laboratory of Experimental Intensive Care and Anesthesiology, Academic Medical Center, University of AmsterdamAmsterdam, Netherlands; ^4^Department of Medical Microbiology, Academic Medical Center, University of AmsterdamAmsterdam, Netherlands

**Keywords:** pandemic, exhaled breath, systems biology, diagnosis, metabolomics, metabolite profiling

## Abstract

Pandemics caused by novel emerging or re-emerging infectious diseases could lead to high mortality and morbidity world-wide when left uncontrolled. In this perspective, we evaluate the possibility of integration of global omics-data in order to timely prepare for pandemics. Such an approach requires two major innovations. First, data that is obtained should be shared with the global community instantly. The strength of rapid integration of simple signals is exemplified by Google’s^TM^ Flu Trend, which could predict the incidence of influenza-like illness based on online search engine queries. Second, omics technologies need to be fast and high-throughput. We postulate that analysis of the exhaled breath would be a simple, rapid and non-invasive alternative. Breath contains hundreds of volatile organic compounds that are altered by infection and inflammation. The molecular fingerprint of breath (breathprint) can be obtained using an electronic nose, which relies on sensor technology. These breathprints can be stored in an online database (a “breathcloud”) and coupled to clinical data. Comparison of the breathprint of a suspected subject to the breathcloud allows for a rapid decision on the presence or absence of a pathogen.

## RATIONALE

Respiratory tract infections are the primary cause of death by communicable diseases (Lopez et al., 2006). The global burden is estimated to be around 3.7 million deaths yearly. Novel emerging or re-emerging infectious diseases could increase the number of victims substantially, as exemplified by the approximately 50 million deaths in the Spanish influenza pandemic in 1918–1919 (Taubenberger and Morens, 2006). Subsequent influenza pandemics and the emergence of novel animal-origin influenza (H5N1, H7N9) and coronaviruses (SARS-CoV, MERS-CoV) that cause severe infections in humans illustrate a continuous and ongoing threat of new pandemics. Intensive farming and changing climate enhance the likelihood of (zoonotic) transmission of animal-origin pathogens to humans and subsequent evolution of such pathogens to efficient infection of – and transmission between humans. Globalization, migrations and intensive tourism further increase the chance of rapid spread of such agents (Jones et al., 2008).

Since infectious disease outbreaks typically emerge unexpectedly and can advance swiftly, rapid detection of (re)-emerging pathogens is of utmost importance. Rapid detection allows for optimal preparation on the level of individuals (e.g., early recognition, quarantine and swift start of adequate treatment of individual patients), on the level of populations (e.g., fast vaccination and other preventive measures), and on the level of organizations (e.g., timely preparation and education of hospital personnel, adequate distribution of therapeutics and medical equipment, and preparation of research infrastructures), thereby hopefully limiting burden caused by each novel rapidly spreading disease.

The two most important challenges are timely recognition of infected individuals and sufficient and timely monitoring of global spread of an outbreak. The first step to optimal preparation may therefore be earlier recognition of infected individuals and global availability of data on spread of outbreaks. In this perspective, we describe a novel vision of how pandemics could be monitored in the future, using global omics-data. We will use influenza as an example as this has been an important causative infection in the past and is likely to cause successive pandemics in the near future.

## THE STATE OF THE ART FOR INFLUENZA DIAGNOSIS AND TREATMENT

As conventional diagnostic methods such as viral culture and detection of antigens or antibodies have limitations due to low sensitivity and delay in time, the officious gold standard for laboratory diagnosis is detection of viral nucleic acids by reverse-transcriptase polymerase chain reaction (RT-PCR; George, 2012). Using this highly sensitive technique, minute amounts of virus can be detected and influenza virus subtypes can be differentiated in less than a few hours. The obvious disadvantages are that skilled personal is needed to perform the tests and that they may not be available during evenings, nights and weekend. This potentially causes delay in the diagnosis of an influenza infection in individual patients (Writing Committee of the WHO Consultation on Clinical Aspects of Pandemic (H1N1) 2009 Influenza et al., 2010).

Especially in case of severe illness, any delay is unwanted as it could hamper timely and life-saving measures and treatment (Writing Committee of the WHO Consultation on Clinical Aspects of Pandemic (H1N1) 2009 Influenza et al., 2010; Ryoo et al., 2013). Of course, one could decide to quarantine and treat every critically ill patient who presents with influenza-like symptoms empirically (Writing Committee of the WHO Consultation on Clinical Aspects of Pandemic (H1N1) 2009 Influenza et al., 2010). Potential side-effects of treatment and high costs associated with quarantine, however, are arguments against such “unselected” treatment, especially on a large scale.

In the scenario of a pandemic with new emerging or re-emerging infections, every delay in adequate diagnosis halts precautionary measures to protect the uninfected individuals. These individuals could be vaccinated, if possible, which may (partially) protect them against the virus when being carried sufficiently ahead of time. Adequate vaccination may also limit further spread of the virus under specific circumstances, as vaccinated individuals not only stay healthy but also will not become contagious themselves. On the level of organization, timely detection allows for preparation and education of hospital personal and distribution of therapeutics and equipment to the desired location.

## GLOBAL RECOGNITION OF FLU THROUGH INTEGRATION OF SIGNALS

Equally important to the time to diagnostic test results is the time to global availability of these data, especially if preparation at the population level and global organization for pandemia prevention is a goal. A system that obliges clinicians to report new cases of severe respiratory viral infections, called the public health response, is available for the clinical suspicion of specific pathogens but this requires a pro-active effort of doctors. Automated integration of test results through an online platform would allow for real-time surveillance. This approach is nicely illustrated by “Flu Trend” in Google^TM^ (http://www.google.org/flutrends/). Using online search engine query data such as “influenza complication” and “flu remedy,” Google^TM^ is able to detect epidemics of respiratory viruses (Ginsberg et al., 2009). This method could predict the incidence of influenza-like illness 1 week before the Center of Disease Control (Ginsberg et al., 2009). However, as the input data for this model are not specific for influenza infection, this tool is helpful for influenza-like illness but probably not sufficient for monitoring the spread of a specific strain of the influenza virus. To capture this complexity, more specific viral signals should be investigated.

## SYSTEMS BIOLOGY AND “OMICS” TECHNOLOGIES

Search engine queries rely on the phenotypic presentation of people with symptoms of influenza-like illnesses. Symptoms are non-specific results of physiological, cellular and molecular changes in the body that occur during viral infection. Systems biology aims to integrate the signals from all these levels into an understanding of the complete system (Josset et al., 2013). Following this philosophy, several “omics” technologies have been developed to measure the molecular landscape in an integrative fashion within one domain. “Genomics” can be used to study genetic risk factors for disease susceptibility of the host (Keynan et al., 2013) and for understanding of the pathogenicity of the pathogen in this context (Kash, 2009). Analysis of mRNA using “transcriptomics” potentially allows for simultaneous measurement of the expression of 10s of 1000s genes. Transcription research allows for rapid testing (e.g., in the order of hours) and provides more information on functionality than genomics alone. However, proteins are ultimately responsible for the function of cells. “Proteomics” has therefore the potential to uncover important interactions between the virus and the host. Studies show that influenza induces rapid changes in the host transcriptome and proteome (Liu et al., 2012; Pommerenke et al., 2012), as soon as 1 h after infection (Cheung et al., 2012). These very early temporal changes after infection are also observed at the metabolite level (Lin et al., 2012). “Metabolomics” is “the global assessment of endogenous metabolites within a biologic system and represents a snapshot-reading of gene function, enzyme activity and the physiological landscape” (Serkova et al., 2011). Treatment of influenza induces many metabolic changes that can be traced back to specific pathways (Lu et al., 2012).

## “OMICS” TECHNOLOGIES FOR GLOBAL INFLUENZA SURVEILLANCE

The systems biology approach has the potential to increase understanding of the spread of a pandemic and the adaptations that viruses undergo meanwhile, as exemplified recently in outstanding research on the influenza virus reservoir in birds (Huang et al., 2013). The major problem with these technologies for monitoring is that they are very time-consuming and expensive, thus conclusions can only be drawn after the pandemic has ended, which is obviously too late. As such, they may only be sufficient for research on the pathogenesis of a pathogen responsible for the pandemic of interest. However, the unbiased approaches of systems biology can be used for unsupervised previsions about disease spreading if this information could be obtained rapidly and at the bedside.

## FOCUS ON EXHALED BREATH

Exhaled breath of infected individuals contains aerosols filled with influenza viruses, mostly present in coarse particles (<5 μm; Milton et al., 2013). This in fact is an important route for the virus to spread. Breath also contains thousands of volatile organic compounds (VOCs), metabolites in gas-phase produced by both physiological and pathophysiological processes (Pauling et al., 1971; Moser et al., 2005). Pulmonary infection, inflammation and oxidative stress may alter the concentration of certain VOCs in exhaled breath (Bos et al., 2013a,b). VOC-patterns identified by smell have been used to diagnose disease and intoxication for ages (e.g., scent of acetone in uncontrolled diabetes; Manolis, 1983). Thus, influenza diagnosis based on exhaled breath analysis could take two forms: detection of aerosols with viral RNA, or an influenza-specific VOC-patterns.

So far, both these methods have relied on relatively time-consuming methods, RT-PCR and gas-chromatography coupled to mass-spectrometry, respectively. Rapid technological innovation in sensors, however, allows for detection of these signals using re-usable, rapid and easy nanosensor arrays. For example, a silicon nano-wire sensor device would allow influenza detection in half the time of RT-PCR in the clinical setting (Shen et al., 2012). Devices using sensor-based detection of VOCs are called “electronic noses,” following their apparent similarities to olfaction (Röck et al., 2008). Electronic noses integratively capture complex VOC mixtures using an array of different sensors (Röck et al., 2008). Sensors have individual sensitivities and specificities for multiple VOCs. The composite signal of all sensors can be analyzed using pattern-recognition algorithms. Electronic nose analysis of breath results in a unique fingerprints of exhaled metabolites, called “breath-prints.” This allows rapid identification, recognition and comparison of VOC mixtures. Thereby, these breath-prints can be used for diagnostic and monitoring purposes, which do not require identification of individual molecular constituents. Breath-prints have found to be different in a wide range of respiratory diseases (Hockstein et al., 2005; Machado et al., 2005; Fens et al., 2009, 2010).

## MONITORING OF MALODOR USING AN ELECTRONIC NOSE

**FIGURE 1:**
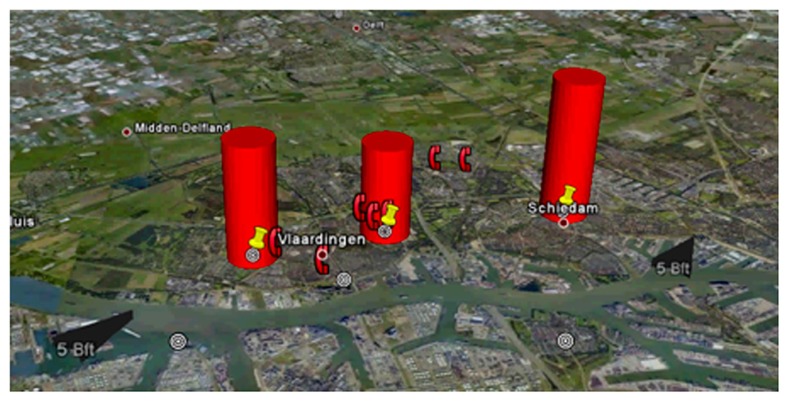
**Odor signatures in the harbor of Rotterdam.** The odor signature disseminates in the direction of the wind leading to increased complaints of inhabitants (telephone symbols on the figure), with permission of Simon Bootsma.

Electronic nose technology is not sufficiently mature for widespread application in clinical practice. However, we can look at other applications to glance at the possibilities for global monitoring using this technology. In the port of Rotterdam, the Netherlands, with 10 km × 10 km one of the largest ports in the world, odor nuisance is a major problem. 30 metal oxide sensor based electronic noses were installed throughout the portal area, to monitor odor emissions in order to timely prevent nuisance (Bootsma and Milan, 2010; Milan et al., 2012). After a training period in which sensors were learned to recognize “malodor,” the electronic noses were able to recognize more than 90% of the reported odor complaints in advance. Based on comparison between fingerprints of the recognized odor and an online database of previous events (an “odor-cloud,” in line with the popular expression for an online virtual server application) the most probable chemical characteristics of the scent can be estimated (Apostulou, 2012). Combined with the temporal findings in different sensors and the direction and speed of the wind, the most probable source of pollution can be identified (**Figure 1**). This approach has allowed for prevention of the development of odor nuisance and environmental pollution by refineries, but also passing cargo ships.

## MONITORING OF EXHALED BREATH USING ELECTRONIC NOSES

**FIGURE 2:**
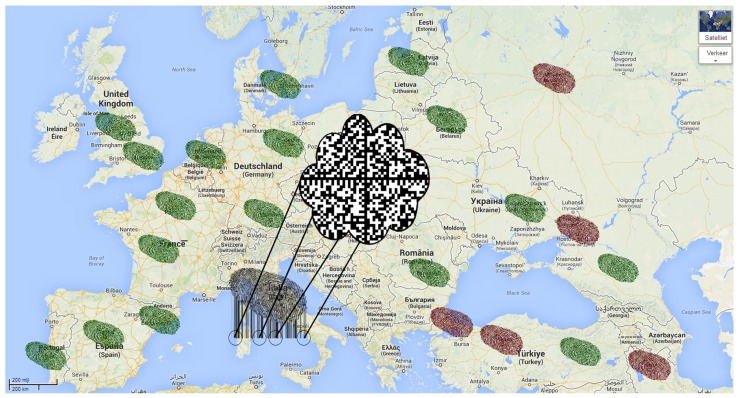
**Future perspective of integration of global omics signals.** There is a centralized database containing molecular fingerprints of past cases, the “breath-cloud” (middle of the picture). Green fingerprints represent suspected cases that were found to be most similar to the non-infected profile. Red fingerprints represent cases that were most similar to the infected profile. There is a new suspected case in Italy, the molecular signature is now compared to the profiles in the breath-cloud.

The parallels between monitoring of the spread of malodor in industrialized areas and of viral infections are striking. In both situations, the timing of the event is unknown, which requires continuous surveillance, and source identification is necessary for early intervention. In the case of exhaled breath analysis, the source cannot be identified with the direction of the wind, but by movement of hosts or patient populations. Therefore, we postulate that exhaled breath tests can best be positioned in places where large groups of people assemble for traveling. One could think of airports, train stations or border control. Here, a very sensitive test may identify infected individuals who are to contribute to the global spread of the pathogen. Importantly, the technique should be high-throughput and the result should be available instantaneously. Thus, in line with environmental surveillance, exhaled breath analysis of patients would allow for the construction of an online database with previously observed breath-prints (a “breath-cloud”; **Figure 2**). Linked with the clinical characteristics of these patients, an exhaled breath pattern for influenza infection can be identified and subsequently used for characterization of new patients. When the clinical information of these patients is known, the breath-cloud can be updated and identification can be improved, allowing for repeated, cyclic improvement of the diagnostic algorithm. There are important differences in the type and concentration of VOCs in environmental and breath analysis. In the environment, the molecules of interest are mostly present in parts-per-million concentration, in contrast to 10s to 100s parts-per-billion in breath. This means that sensors for breath monitoring need to be more sensitive. The VOCs of interest are mostly sulfur-containing and cyclic compounds in studies on maladour but breath research is not limited to those. Therefore it is anticipated that the sensor array ought to be larger and more versatile in breath analysis.

## REQUIRED STEPS FOR INTEGRATING THE METHODOLOGY

Several steps are needed to accomplish the above-suggested approach:

• The VOCs that can be used for early diagnosis of a viral infection need to be identified. A very sensitive diagnosis is a first requirement for global screening as the goal is to isolate a small potion of the population while maintaining a very high negative predictive value.

• An appropriate array of sensors needs to be assembled. These arrays need to contain several sensors that are designed to react selectively with the previously identified VOCs and a wide variety of general, semi-selective (cross-reactive) sensors (Konvalina and Haick, 2013). The former are used for specific identification of a viral infection but are prone to changes in virus induced exhaled breath profile that may occur over time, due to viral mutations or phenotypic changes. The latter allows for plasticity of the diagnostic algorithm by reacting with unselected VOCs.

• An online centralized database, were to breath-prints are uploaded, instantly should be created. Thus, the electronic noses should be connected to the Internet, which allows for synchronization with the breath-cloud.

• The electronic noses should be readily available in the areas where the first signals of an outbreak are visible.

## OTHER TECHNOLOGIES

Exhaled breath analysis by electronic nose may not be the only technology that is continuously available and provides a direct test result. Any technology that gives a rapid result can be used for the same purpose. All signals can be used complementary by uploading them together to the same online database. Here a pattern recognition algorithm can treat these signals similar and will select only the most discriminative, updating prior beliefs with every additional information that becomes available. The development of bedside PCR machines is exciting in this respect as these could allow for very accurate and rapid detection of viral RNA (Centers for Disease Control and Prevention, 2013). Further miniaturization and optimization toward a “lab on a chip” will further improve the time to result and bedside use (Sun et al., 2011; Song et al., 2012).

## CONCLUSION

To conclude, there is a need for rapid diagnosis of specific infections (including but not restricted to outbreaks of influenza), especially during a pandemic. High-throughput chemical profiling of patient material could provide a fast and objective means to diagnose patients. At this moment, portable electronic nose technology is a good example of how these infections can be captured rapidly at the bedside and can be shared with the world in the form of a “breath-cloud.” In principle, any technology that provides test results rapidly could attribute to this online database. Pattern recognition software can subsequently be used to diagnose new suspected cases based on previous profiles from patients all over the world.

## Conflict of Interest Statement

The authors declare that the research was conducted in the absence of any commercial or financial relationships that could be construed as a potential conflict of interest.
